# Prevalence of hazardous alcohol consumption and evaluation of associated factors in university students

**DOI:** 10.1007/s00127-024-02680-8

**Published:** 2024-05-08

**Authors:** Ahmet Ay, Cüneyt ÇAM, Ali KILINÇ, Muhammed Fatih ÖNSÜZ, Selma METİNTAŞ

**Affiliations:** 1 Department of Public Health, Faculty of Medicine, Eskisehir Osmangazi University, Eskisehir, Turkey; 2 Department of Public Health, Mardin Health Directorate, Mardin, Turkey; 3 Department of Public Health, Beysehir Health Directorate, Konya, Turkey

**Keywords:** AUDIT, Epidemiology, Hazardous alcohol consumption, University students, Sociodemographic factors

## Abstract

**Purpose:**

The aim of the study was to determine the prevalence of hazardous alcohol consumption (HAC) according to gender among university students and associated factors.

**Methods:**

This is a cross-sectional study conducted on undergraduate students. We used a stratified sampling technique to represent 26036 students from all grade levels and 11 faculties, and the survey was administered to 2349 undergraduate students. The prevalence of HAC was determined with the Alcohol Use Disorders Identification Test (AUDIT). HAC was defined as getting 8 points or more from the AUDIT. Multivariate logistic regression analyses were performed to examine HAC related factors in both genders.

**Results:**

In this study, 53.2% of the participants were male. The prevalence of HAC in the study group was 13.5% and prevalence of lifetime drinker was 65.3%. In males; those whose fathers [OR = 1.72; 95% CI: (1.17-2.52)], mothers [1.49; (1.02-2.18)], close friends [2.42; (1.28-4.60)] drink alcohol and smoking [3.16; (2.09- 4.77)], use illicit substance [2.35; (1.66-3.34)], have mental health problems [1.65; (1.04-2.62)] were more likely to report HAC. Meanwhile in females, those whose fathers [OR = 1.92; 95%CI: (1.03-3.57)], close friends [5.81; (1.73-19.45)] drink alcohol and smoking [4.33; (2.31-8.15)], use illicit substance [4.34; (2.34-8.06)] have mental health problems [3.01; (1.67-5.43)] were more likely to report HAC.

**Conclusions:**

HAC prevalence is high among university students. The risk of HAC increases with the use of alcohol in family and circle of friends, smoking, illicit substance use and mental health problems. The factors associated with the risk of HAC in both genders are similar.

## Introduction

In many cultures, drinking alcohol is seen as a pleasurable activity that has numerous cultural and social aspects in addition to personal biological and psychological components [1, 2]. As it is legal, unlike other addictive substances, its negative effects are as widespread as its consumption, making it a serious public health problem [2]. The harmful use of alcohol has been linked to the risk of more than 200 diseases and injuries, including non-communicable diseases, some cancers, mental and behavioural disorders, accidents and violence [2, 3]. According to the World Health Organisation’s (WHO) Global Status Report on Alcohol and Health 2018, an estimated 5.3% of deaths worldwide (approximately 3 million deaths per year) and 5.1% of disability-adjusted life years (DALYs) are attributable to alcohol [2]. The proportion of all deaths caused by alcohol rises to up to 13.5% in the 20to 39 age group [4].

According to the WHO, 44.5% of the world’s population drank alcohol last year [2]. It is estimated that around 2.3 billion people in the world consume alcohol and that on average 6.4 L of pure alcohol are consumed per capita [4]. According to the WHO, Turkey, where 89.1% of the population does not drink alcohol, appears to be the country with the lowest alcohol consumption in the WHO European region, with an average per capita consumption of pure alcohol of around one third of the global average. Although this amount is far below the global average, the amount of pure alcohol per capita of alcohol drinkers in Turkey is estimated at 28.5 L, while the average amount of pure alcohol per capita in the European region, where alcohol consumption is highest, is 17.2 L [2]. According to the 2015 OECD (Organisation for Economic Co-operation and Development) report Tackling Harmful Alcohol Use Economics and Public Health Policy, alcohol is consumed intensively by the 20% of the population who drink the most [5]. This fact emphasises how important the patterns of alcohol drinking are in addition to the quantity of alcohol consumed.

Alcohol drinking patterns can occur in a spectrum ranging from hazardous and harmful alcohol consumption to heavy and intensive alcohol consumption and alcohol use disorders including alcohol dependence [6, 7]. The prevalence of alcohol use disorders in the European region for the general population aged 15 years and older was reported to be 8.8%, while in Turkey it is estimated to be 1.7% in women and 8.1% in men [2]. azardous alcohol consumption (HAC) is defined as a repetitive drinking pattern that does not fulfil the criteria for alcohol use disorder or dependence but carries the risk of causing harmful consequences for the drinker or others [6]. Epidemiological studies show that most physical, social and psychological problems associated with alcohol consumption occur in drinkers who are not defined as dependent [6].

The period between the ages of 18 and 25, also known as emerging adulthood, lies between adolescence and early adulthood and is a developmental transition period with unique characteristics [8]. During this period, which also includes the university education phase, different lifestyles and values are learnt and experienced. It is known that risky behaviours can be tolerated and even encouraged during this period as freedom and peer contact increase and social control decreases [9]. It is known that lifetime prevalence and related problems with alcohol drinking, smoking and illicit substance use peak in young adulthood [9, 10]. Epidemiological studies have shown that the frequency and quantity of alcohol drinking and HAC patterns are more common in university students than in their non-university peers [11–14]. Due to the social acceptance of alcohol drinking among university students as part of the university culture, HAC patterns among university students may be underestimated and overlooked [15].

Although patterns of alcohol drinking vary from country to country and from culture to culture, gender is one of the most important sources of heterogeneity within populations in terms of alcohol drinking and related problems [16, 17]. It is known worldwide that men are more prone to alcohol consumption, consume more alcohol than women and are more affected by the subsequent negative consequences [1, 2, 16]. However, it has been reported that the gender gap in indicators of alcohol drinking and associated harms tends to narrow significantly, especially in the young adult age group [18]. Although the same situation was observed in studies conducted among university students, it is noteworthy that high levels of alcohol consumption were found in both genders [19, 20]. In many countries, hazardous or harmful alcohol consumption patterns remain the biggest problem of substance misuse during university life [20, 21].

The aim of the study was to determine the prevalence of hazardous alcohol consumption (HAC) according to gender among university students and associated factors.

## Methods

This study is a cross-sectional study performed on undergraduate students at a university in Eskisehir, a province in the north-west of Turkey in the Central Anatolia region, during the 2019–2020 academic year, i.e. before the COVID-19 pandemic. The province of Eskisehir is home to over 70 thousand students from three universities.

Eskisehir Osmangazi University (ESOGU) was founded in 1970 and today has 12 faculties, 2 colleges, 5 vocational schools, 4 institutes and 32 application and research centres. The research population consists of 26,036 students receiving formal education in undergraduate programmes of 4 or more years [22, 23]. The sample size was calculated with a 95% confidence interval and a 2% margin of error to 2,198 individuals, assuming a 50% prevalence of HAC. Each faculty was considered as a stratum and the number of students to be included in the sample from each faculty was determined taking into account the weights of the strata. To account for loss of data due to incomplete forms and inappropriate responses, it was planned to include 2,400 students in the study.

Administrative and ethical approval (E122483, 22.10.2019) was obtained to conduct the study. Appropriate days and times for data collection were determined together with the faculty administrators, and students were required to assemble in their classrooms prior to data collection. The prepared questionnaires were completed by the students under observation in about 10–15 min. The study group consisted of 2349 students who agreed to participate in the study and answer the questionnaires.

A questionnaire was created for data collection in the study using the literature [20, 24, 25]. The questionnaire consisted of two parts. The first part included some sociodemographic characteristics (age, gender, place of residence, income situation, etc.) and some factors thought to be related to HAC (alcohol consumption by parents or close friends, smoking and illicit substance use, etc.). The second part of the questionnaire consisted of the Alcohol Use Disorder Identification Test (AUDIT).

The AUDIT, developed by WHO and Babor et al., consists of 10 questions including alcohol drinking habits, alcohol consumption and problems caused by alcohol drinking [26]. The scores achievable with the scale range from 0 to 40, and scores of 8 and above are defined as “HAC” [27]. The scale was adapted into Turkish by Saatcioglu et al. in 2002 [28].

In the study, the alcohol consumption status of the individuals was categorized as follows; those who have experienced alcohol at least once in their lives were classified as “lifetime drinkers”, those who had never consumed alcohol were classified as “lifetime abstainers”, ”, those who stated that they had drunk alcohol at least once in the past year were classified as “current drinkers” and those who stated that they had consumed alcohol but had stopped in the past year were classified as “former drinkers” [29]. .

Students who regularly smoked at least 1 conventional cigarette and heated tobacco products per day were considered as"smokers” [30]. Smoking status was categorized into three groups (never, former or current smoker) based on participants’ responses. Illicit substance use status was categorized into three groups (never, former, or current user) based on participiants’ response on illicit substance. The academic achievement status of the students was evaluated as “lower than average”, “average” and “higher than average” according to their own perceptions. In order to obtain realistic results in the evaluation of illicit substance use, a fake substance named “relactin” was added and 17 individuals who stated that they used this substance were not included in the study [31].

The data were then transferred to the computer and analysed using the SPSS 15.0 (Statistical Program in Social Sciences, SPSS Inc., Chicago, IL, USA). The students were divided into two groups according to their HAC for both genders. In the statistical evaluation of the data, descriptive data were given as number, percentage, mean and standard deviation. In the first step in the statistical evaluation of the data, the relationships between sociodemographic and related factors and HAC were evaluated using Chi-square analysis. The relationships found to be significant in the univariate analyses were then re-evaluated using the Multivariate Logistic Regression model for both gender groups. p values < 0.05 were considered statistically significant.

## Results

Of the 2,349 students who participated in the study, 1,250 (53.2%) were male and 1099 (46.8%) were female. The age of the study group ranged from 18 to 35 years, with a mean (SD) of 21.04 (2.07) years. While 1,147 (48.8%) of the students participating in the study were studying in Natural/Engineering Sciences, 682 (29.0%) were in fourth grade and above. The distribution of the sample by gender and some socio-demographic characteristics is presented in Table 1.

**Table 1:** Sociodemographic Characteristics of the study group

		% (*n*)	Male % (*n*)	Female % (*n*)
**Gender**				
Male		53.2 (1,250)		
Female		46.8 (1,099)		
**Age**				
≤ 19		22.4 (526)	18.3 (229)	27.0 (297)
20–21		42.7 (1,002)	41.5 (519)	43.9 (483)
≥ 22		35.0 (821)	40.2 (502)	29.0 (319)
**Faculty**				
Education Sciences		16.9 (397)	11.0 (137)	23.7 (260)
Natural / Engineering Sciences		48.8 (1,147)	56.1 (701)	40.6 (446)
Health Sciences		14.0 (330)	10.6 (133)	17.9 (197)
Social/Humanities		20.2 (475)	22.3 (279)	17.8 (196)
**Year of study**				
1th		23.9 (561)	22.8 (285)	25.1 (276)
2th		23.3 (546)	22.6 (283)	23.9 (263)
3th		23.8 (560)	23.6 (295)	24.1 (265)
4th and above		29.0 (682)	31.0 (387)	26.8 (295)
**Status of residence**				
With the family		19.7 (463)	18.5 (231)	21.1 (232)
With his friend		62.0 (1,456)	58.3 (729)	66.2 (727)
Alone		18.3 (430)	23.2 (290)	12.7 (140)
**Academic achievement status**				
Lower than average		11.1 (261)	13.0 (162)	9.0 (99)
Average		63.4 (1,490)	61.3 (767)	65.8 (723)
Higher than average		25.5 (598)	25.7 (321)	25.2 (277)
**Income situation**				
Low		8.9 (210)	11.5 (144)	6.0 (66)
Medium		57.5 (1,350)	56.2 (702)	59.0 (648)
High		33.6 (789)	32.3 (404)	35.0 (385)
**Smoking**				
Never smoker		49.2 (1,155)	39.7 (496)	60.0 (659)
Current and former smoker		50.8 (1,194)	60.3 (754)	40.0 (440)
**Illicit substance use**				
Never used		87.5 (2,055)	81.9 (1,024)	93.8 (1,031)
Current and former user		12.5 (294)	18.1 (226)	6.2 (68)
**Physician diagnosed any mental health problem**				
None		90.0 (2,114)	90.7 (1,134)	89.2 (980)
Present		10.0 (235)	9.3 (116)	10.8 (119)

**Graph 1:**
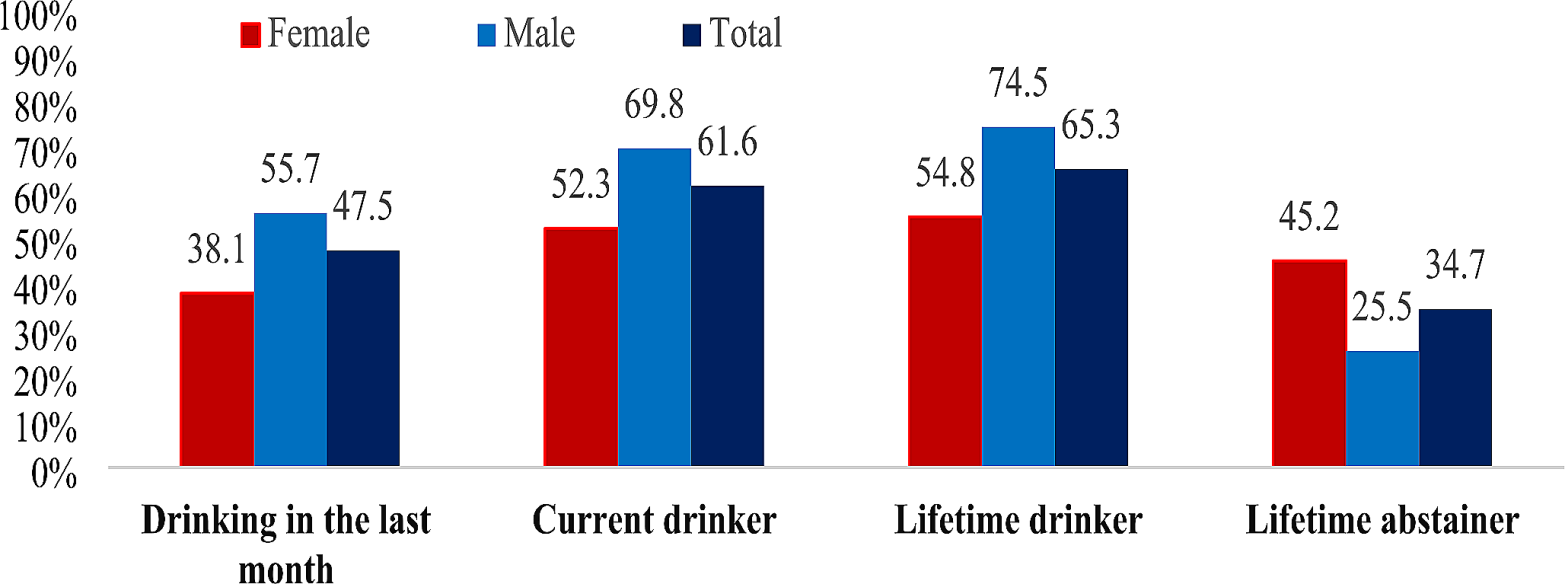
Distribution of alcohol consumption status of the research group by gender

In the study group, 53.2% of female students and 69.8% of male students stated that they current drinkers (*p* < 0.001). While 25.5% of male students reported a lifetime abstinence form alcohol consumption, this prevalence was 45.2% for female students (*p* < 0.001). The distribution of the alcohol drinking status of the students in the study group according by gender is presented in Graph 1.

HAC was detected in 317 (13.5%) students in the study group. The prevalence of HAC was 18.8% in male students and 8.2% in female students. The prevalence of HAC was significantly higher in male and female students who lived alone at home, smokers, used illicit substances, and had any mental health problems. The prevalence of HAC was higher in both gender among those whose mother, father and close friend consumed alcohol. The prevalence of HAC was lower among those with medium income in terms of both genders. Among male students, the prevalence of HAC was higher among those studying in Social/Humanities than those studying in Natural/Engineering and Education Sciences. Among female students, the prevalence of HAC was lower among those studying in Educational Sciences compared to those studying in other faculties. The distribution of the prevalence of HAC according to the socio-demographic characteristics and lifestyle factors of the students is presented in Table 2.

**Table 2:** Distribution of frequency of HAC according to sociodemographic characteristics and lifestyle factors of students

		Male *n* = 1250 (53.2%)	*p*	Female *n* = 1099 (46.8%)	*p*
**HAC**					
Present		227 (18.2)	**< 0.001**	90 (8.2)	**< 0.001**
**Age**					
≤ 19		34 (14.8)	0.051	20 (6.7)	0.225
20–21		86 (16.6)		37 (7.7)	
≥ 22		107 (21.3)		33 (10.3)	
**Faculty**					
Education Sciences		17 (12.4)		5 (1.9)	
Natural / Engineering Sciences		115 (16.4)	**0.009**	46 (10.3)	*p* < 0.001
Health Sciences		28 (21.1)		20 (10.2)	
Social/Humanities		67 (24.0)		19 (9.7)	
**Year of study**					
1th		47 (16.5)	0.788	18 (6.5)	0.219
2th		56 (19.8)		18 (6.8)	
3th		53 (18.0)		22 (8.3)	
4th and above		71 (18.3)		32 (10.8)	
**Status of residence**					
With the family		34 (14.7)	*p* < 0.001	15 (6.5)	*p* < 0.001
With his friend		127 (17.4)		49 (6.7)	
Alone		66 (22.8)		26 (18.6)	
**Academic achievement status**					
Lower than average		37 (22.8)	0.223	10 (10.1)	0.247
Average		131 (17.1)		52 (7.2)	
Higher than average		59 (18.4)		28 (10.1)	
**Income situation**					
Low		33 (22.9)	**0.021**	9 (13.6)	**0.030**
Medium		109 (15.5)		42 (6.5)	
High		85 (21.0)		39 (10.1)	
**Smoking**					
Never smoker		33 (6.7)	*p* < 0.001	14 (2.1)	*p* < 0.001
Current and former smoker		194 (25.7)		76 (17.3)	
**Illicit substance use**					
Never used		139 (13.6)	*p* < 0.001	61 (5.9)	*p* < 0.001
Current and former user		88 (38.9)		29 (42.6)	
**Physician diagnosed any mental health problem**					
None		187 (16.5)	*p* < 0.001	64 (6.5)	*p* < 0.001
Present		40 (34.5)		26 (21.8)	
**Alcohol consumption by the mother**					
None		142 (14.6)	*p* < 0.001	50 (5.8)	*p* < 0.001
Present		85 (30.4)		40 (17.1)	
**Alcohol consumption by the father**					
None		62 (10.5)	*p* < 0.001	20 (3.6)	*p* < 0.001
Present		165 (25.0)		70 (12.8)	
**Alcohol consumption by the close friends**					
None		12 (4.8)	*p* < 0.001	3 (0.7)	*p* < 0.001
Present		215 (21.5)		87 (12.7)	

A multivariate logistic regression model was constructed with the factors influencing HAC in terms of both genders (faculty of education, status of residence, income situation, smoking, illicit substance use status, physician diagnosed any mental health problem, alcohol consumption of mother, father and close friend) in the study group.

According to the results of the logistic model, the risk of HAC among male students increased 3.16 times for smoking [OR = 3.16; 95%CI: (2.09–4.77)], 2.35 times for illicit substance use [2.35; (1.66–3.34)], 1.65 times for any mental health problem diagnosed by a physician [1.65; (1.04–2.62)], 1.72 times for father’s alcohol consumption [1.72; (1.17–2.52)] and 2.42 times for close friend’s alcohol consumption [2.42; (1.28–4.60)]. The risk of HAC among female students was 4.33 times higher for smoking [4.33; (2.31–8.15)], 4.34 times higher for illicit substance use [4.34; (2.34–8.06)], 3.01 times higher for any mental health problem diagnosed by a physician [3.01; (1.67–5.43)], 1.92 times higher for father’s alcohol consumption [1.92; (1.03–3.57)] and 5.81 times higher for close friend’s alcohol consumption [5.81; (1.73–19.45)]. Mother’s alcohol consumption did not show a significant association with HAC in female students, whereas the risk of HAC increased 1.49 times in male students [1.49; (1.02–2.18)]. Faculty of study, status of residence and income situation were found to be unrelated to HAC in both genders. The multivariate logistic regression model analysis of the risk factors associated with HAC in male and female students is presented in Table 3.

**Table 3:** Multivariate logistic regression analysis of risk factors associated with students’ HAC

		Male				Female		
	HAC %	OR	95% CI	*p*	HAC %	OR	95% CI	*p*
**Faculty**								
Education Sciences	12.4	1.00		0.063	1.9	1.00		0.192
Natural / Engineering Sciences	16.4	1.19	0.66–2.15		10.3	2.75	1.02–7.45	
Health Sciences	21.1	1.73	0.85–3.54		10.2	3.21	1.10–9.42	
Social/Humanities	24.0	1.82	0.98–3.40		9.7	2.71	0.93–7.89	
**Status of residence**								
With the family	14.7	1.00			6.5	1.00		0.478
With his friend	17.4	1.23	0.79–1.93	0.557	6.7	1.04	0.53–2.05	
Alone	22.8	1.30	0.79–2.14		18.6	1.48	0.67–3.27	
**Income situation**								
Low	22.9	1.00			13.6	1.00		
Medium	15.5	0.76	0.47–1.23	0.490	6.5	0.71	0.28–1.76	0.463
High	21.0	0.87	0.53–1.44		10.1	0.96	0.38–2.45	
**Smoking**								
Never smoker	6.7	1.00		**< 0.001**	2.1	1.00		**< 0.001**
Current and former smoker	25.7	**3.16**	2.09–4.77		17.3	**4.33**	2.31–8.15	
**Illicit substance use**								
Never used	13.6	1.00		**< 0.001**	5.9	1.00		**< 0.001**
Current and former user	38.9	**2.35**	1.66–3.34		42.6	**4.34**	2.34–8.06	
**Physician diagnosed any mental health problem**								
None	16.5	1.00		**0.032**	6.5	1.00		**< 0.001**
Present	34.5	**1.65**	1.04–2.62		21.8	**3.01**	1.67–5.43	
**Alcohol consumption by the mother**								
None	14.6	1.00		**0.037**	5.8	1.00		0.858
Present	30.4	**1.49**	1.02–2.18		17.1	0.95	0.54–1.67	
**Alcohol consumptionb by the father**								
None	10.5	1.00		**0.006**	3.6	1.00		**0.038**
Present	25.0	**1.72**	1.17–2.52		12.8	**1.92**	1.03–3.57	
**Alcohol consumption by the close friends**								
None	4.8	1.00		**0.007**	0.7	1.00		**0.004**
Present	21.5	**2.42**	1.28–4.60		12.7	**5.81**	1.73–19.45	

## Discussion

In the study, while most of the university students in the study were current drinkers, the prevalence among male students reached worrying levels. While HAC is found in one in five students who current drinker, the gender gap remains. Similar factors had an influence on the HAC of male and female students. Students’ mental health status, smoking and illicit substance use habits, and the drinking habits of their social and close circle were associated with the prevalence of HAC among students.

In the study, about three out of five students (61.6%) were current drinkers. While the prevalence among men was 69.8%, 52.3% of women were current drinkers. An international study on the alcohol use of university students from 21 countries found that, despite large differences between countries, the prevalence of current drinkers ranged between 29 and 95%, with a higher prevalence among men [32]. In various studies, the prevalence of current alcohol consumption among university students; while it is 77.6% in the USA [33], 90% in Australia [34], 92% in the UK [35] and it rises to 95% in Northern European countries in particular [32]. It is also reported that the gender gap in the prevalence and amount of alcohol consumption in Northern European countries and the United Kingdom have narrowed or even disappeared [36]. Stock et al. in their study of university students from various European countries, it was reported that the frequency of occasionally or not drink alcohol was highest among Turkish students [37]. Various studies conducted in Turkey have shown that the prevalence of current drinkers among university students varies between 34.1 and 59.9%, with a higher prevalence among men [38–42]. The lifetime prevalence of alcohol among Turkish university students has been reported to vary between 46.1 and 76.2/63.3% [38, 40, 43–45] and in our study it was found to be 65.3%. In Turkey, where cultural diversity is high, alcohol drinking is more common among university students in the west of the country due to the influence of socio-economic and socio-cultural regional differences [43, 46], although the prevalence of current drinkers in the west is over 50% [41, 42]. The regional characteristics of the city in which the study was conducted, the socialisation opportunities for students and the social acceptance of alcohol drinking may have affected the prevalence of drinking among the students in the study.

Although most students in the study were current drinkers, HAC was found in 13.5% of students, and the prevalence increased to 22% among students who were current drinkers. HAC was more common among male students (18.2%) than female students (8.2%). Although difficult to compare the results obtained in this study with previous studies due to the different definitions of risky/hazardous alcohol consumption, the Cut-down, Annoyed, Guilty and Eye-opener (CAGE) questionniare and the AUDIT screening tool are the two most commonly used standardised measurement instruments used in the screening of problematic alcohol consumption (risky or hazardous patterns of alcohol consumption) [20]. In the study conducted by Cooke et al. among university students from various European countries, it was found that 37% of students were positive on AUDIT (8 or more), with a higher prevalence in men [47]. While a gender gap was found in various studies [34, 48, 49] using the AUDIT, HAC is 11.4% in Ethiopian university students [50], 16.9% in Spanish university students [49] and 34% in Australian university students [34], while reaches 60% in university students form UK [35, 47] and Northern European countries [19, 47]. However, some studies using the AUDIT have reported that HAC is much more common among university students in Northern European countries and UK and that the gender gap is closing [20, 51].

In various studies using the AUDIT in Turkey, the prevalence of HAC among university students, although higher in the west, varies from 4.5 to 22.2%, and it has been reported to be more common in males [39, 42, 44, 52]. In studies in which problematic alcohol consumption among Turkish university students is assessed with CAGE (2 or more), the prevalence of risky alcohol consumption/drinking varies between 7.4% and 15.3% [38, 40, 43, 45]. In the study by Sebena et al. in which risky consumption was assessed with CAGE (+ 2 points) among university students from various European countries, it was reported that the prevalence was between 11.8% and 22.1% [53]; while in another international study the lowest prevalence of risky alcohol consumption was found among Turkish female students (8%) [37]. Although the prevalence in the study did not reach the level of European and US university students, around one in five students who consumed alcohol met the HAC threshold. But even though problematic alcohol consumption such as HAC is not widespread among university students, it is known that the majority of students are harmed by their own consumption or that of other students [34, 54]. Gender differences in risky/hazardous alcohol consumption are expected to be greatest in countries where there are restrictions on women’s behaviour and gender inequalities in daily life [55]. The gender gap in HAC among the students in the study could be due to socio-cultural factors, such as the fact that alcohol consumption by women in Turkey is less socially accepted compared to men, that women are more sensitive to social acceptance and that men are more competitive in risky behaviour.

Although adolescence and emerging adulthood are risky phases for starting substance use, smoking and alcohol use generally begin before the use of illicit substances [56]. In our study, around one in two students stated that they smoker and one in ten students reported that they had experience with illicit substances use. While smoker was the factor that increased HAC among male students the most (3.2 times), smoking and illicit substance use increased HAC among female students more than four times. It is known that there is a complex relationship between tobacco and alcohol use which depends on both social and pharmacological factors [57] and that smoking in emerging adulthood is also prospectively associated with higher alcohol consumption [58]. It has also been shown that the co-occurence of smoking and drinking [59] and even non-daily smoking increases the risk of HAC [60]. In a systematic review of studies conducted on university students from various European countries, a strong association was found between the frequency and quantity of alcohol drinking and smoking, while students with high alcohol consumption were reported to be more likely to use illicit substances [36]. Various studies have shown that the prevalence of HAC is higher among university students who smoking and/or illicit substance use [19, 35, 50, 61]. A similar relationship was found in various studies among university students in Turkey [42, 44, 52]. Although risky behaviours are common among emerging adulthood, it is known that smoking and alcohol, which are legal and easily accessible substances, increase the risk of abuse of each other and other illicit substances. The possibility of other substance use problems in students with HAC should not be ignored.

Young adults are similar to their close friends or peers in terms of alcohol use, and it is known that peer norms have a strong influence on risky behaviours such as alcohol drinking [62]. In the study, although alcohol drinking by close friends is one of the factors influencing HAC among students of both genders, it is the factor that increases the risk of HAC the most among female students (5.8 times). University students are more influenced by the alcohol drinking they perceive among their close friends than by the drinking of their other peers at the university [63]. Our result is in line with various studies that show that the alcohol drinking of close friends or the circle of friends has an influence on the risky/hazardous alcohol consumption of students [36, 44, 45, 50, 54]. Furthermore, emerging evidence suggests that the influence of peers on an individual’s alcohol drinking in a socio-cultural context can become the norm not only in adolescence and young adulthood, but also in adult [64]. Given the homogeneity of peer groups of university students, it is likely that students have close friends with similar alcohol drinking patterns, which may make the social environment conducive to alcohol drinking. In the study, however it is not possible to make a statement about whether the students’ HAC is influenced by the alcohol drinking of their close friends or whether they choose their close friends based on similarities in their alcohol drinking paterns.

Emerging evidence shows that the alcohol drinking patterns of parents has less influence on the problematic alcohol consumption of adolescents and young adults compared to the influence of peers [62, 65]. In the study found that parental alcohol drinking increases the risk of HAC in students. While the alcohol drinking of both parents has an influence on HAC of male students, the relationship between HAC of female students and the alcohol drinking of the mother disappeared in the multivariate logistic model. It is to be expected that the attitudes and behaviours of parents in relation to alcohol will serve as a reference for the alcohol drinking patterns of university students who take on the role of adults [16, 66]. Although various studies conducted among university students have shown the opposite [38, 43, 50], many studies have reported that parents’ alcohol drinking pattern has an influence on students’ risky/hazardous alcohol consumption [44, 67, 68]. Carey et al. emphasised in their meta-analysis that although the presence of a family history has a minimal influence on alcohol drinking among students, the presence of a family history can increase the risk of students developing alcohol-related problems [67]. Although the result of our study is consistent with the literature, the fact that we could not find any influence of maternal alcohol drinking on the HAC of the female students could be due to the small number of drinker mothers who, according to the students in the sample.

Finally, another important variable that increases the risk of HAC among the students in the study is the presence of a diagnosed mental health problem. Our study found that HAC was 1.6 times more common in male students with mental health problems and 3.0 times more common in female students. In addition to emerging adulthood a risk period for mental health problems [69], but starting university can also pose a risk to people’s mental health during this time [70]. Poor mental health is known to fueling the risk of drinking and problematic alcohol consumption [71], and the emphasis on the prevalence and severity of mental health problems among university students should also be considered in this regard [72, 73]. Various studies conducted among university students also support our conclusion by showing a link between mental health problems and risky/hazardous alcohol consumption [42, 48, 52, 53, 61]. Among university students, the fact that health-risk behaviours tend to cluster among students with poor mental health [74] may explain the association in our study.

### Strengths and weaknesses

The strengths of the study are the use of an internationally recognised screening tool whose validity and reliability have been previously verified to objectively detect HAC among university students and the use of a large sample of university students with a stratified sampling method. Although the variations in the definition of risky/hazardous alcohol consumption in studies conducted among university students in Turkey and worldwide lead to differences in prevalence, the results can be considered comparable in terms of the factors that may be related to it. In our study, when defining HAC in the AUDIT screening tool, we favoured the threshold of AUDIT 8 points and above, which has been frequently reported in previous studies, to facilitate the comparability of our results. However, the interpretation of our results could have changed if we had chosen to define HAC using other thresholds. The cross-sectional design of the study does not show a causal relationship, and the fact that the sample is limited to a single university makes it impossible to generalise the results. Another limitation of the study is the fact that the information provided by the students is based on recall and self-reporting. The fact that students who did not agree to participate in the study and absent students were not included in the sample may have led to a slight underestimation of HAC. We also have no reason to believe that those who were not in class on the day of the survey and did not respond to the survey had less HAC. This study is one of the rare studies to examine risky/hazardous alcohol consumption among university students, which is a current issue in many countries, among Turkish university students using a large sample, although it is limited to a single university.

## Conclusion and suggestions

In this study, age, years of study, income situation, status of residence, faculty, and academic achievement of students were found to be no significant difference to report HAC in both genders. In the study, the prevalence of HAC, which entails social, physical and psychological risks in the short and long term, did not reach the level of European and US students. However, while three in five university students are current drinkers, one in five students who drink have HAC, indicating that the prevalence is not low. While the prevalence among male students is more notable, the gender gap remains among Turkish students. However, similar factors had an impact on the HAC of male and female students. Students’ mental healths, smoking and illicit substance use habits, and the drinking habits of their social and close circle increased the risk of HAC among students. Given the negative consequences of alcohol drinking, screening for problematic alcohol consumption among university students, an at-risk group, is of great importance for planning effective prevention and education measures and for early detection. We emphasise that other substance use problems may also exist when planning interventions to address student alcohol use and that it is important not to ignore mental health and perceived alcohol drinking among peers. In addition, intervention programmes involving parents can be useful to prevent risky/hazardous alcohol consumption among university students and raise their awareness.
